# Serotonin receptor‐mediated vasorelaxation occurs primarily through 5‐HT_4_ activation in bovine lateral saphenous vein

**DOI:** 10.14814/phy2.16128

**Published:** 2024-06-30

**Authors:** Ronald J. Trotta, David L. Harmon, James L. Klotz

**Affiliations:** ^1^ Department of Animal and Food Sciences University of Kentucky Lexington Kentucky USA; ^2^ Forage‐Animal Production Research Unit USDA‐ARS Lexington Kentucky USA

**Keywords:** 5‐hydroxytryptamine, BIMU 8, cattle, G protein‐coupled receptor, ruminant

## Abstract

To better understand mechanisms of serotonin‐ (5‐HT) mediated vasorelaxation, isolated lateral saphenous veins from cattle were assessed for vasoactivity using myography in response to increasing concentrations of 5‐HT or selective 5‐HT receptor agonists. Vessels were pre‐contracted with 1 × 10^−4^ M phenylephrine and exposed to increasing concentrations of 5‐HT or 5‐HT receptor agonists that were selective for 5‐HT_1B_, 5‐HT_2B_, 5‐HT_4_, and 5‐HT_7_. Vasoactive response data were normalized as a percentage of the maximum contractile response induced by the phenylephrine pre‐contraction. At 1 × 10^−7^ M 5‐HT, a relaxation was observed with an 88.7% decrease (*p* < 0.01) from the phenylephrine maximum. At 1 × 10^−4^ M 5‐HT, a contraction was observed with a 165% increase (*p* < 0.01) from the phenylephrine maximum. Increasing concentrations of agonists selective for 5‐HT_2B_, 5‐HT_4_, or 5‐HT_7_ resulted in a 27%, 92%, or 44% (*p* < 0.01) decrease from the phenylephrine maximum, respectively. Of these 5‐HT receptor agonists, the selective 5‐HT_4_ receptor agonist resulted in the greatest potency (−log EC_50_) value (6.30) compared with 5‐HT_2B_ and 5‐HT_7_ receptor agonists (4.21 and 4.66, respectively). To confirm the involvement of 5‐HT_4_ in 5‐HT‐mediated vasorelaxation, blood vessels were exposed to either DMSO (solvent control) or a selective 5‐HT_4_ antagonist (1 × 10^−5^ M) for 5‐min prior to the phenylephrine pre‐contraction and 5‐HT additions. Antagonism of the 5‐HT_4_ receptor attenuated the vasorelaxation caused by 5‐HT. Approximately 94% of the vasorelaxation occurring in response to 5‐HT could be accounted for through 5‐HT_4_, providing strong evidence that 5‐HT‐mediated vasorelaxation occurs through 5‐HT_4_ activation in bovine peripheral vasculature.

## 1 INTRODUCTION

Consumption of ergot alkaloids found in wildtype endophyte‐infected (*Epichlöe coenphialum*) tall fescue (*Lolium arundinaceum*) can lead to fescue toxicosis, a form of ergotism in ruminant livestock species (Bush et al., 1982; Lyons et al., 1986; Strickland et al., 2011). Increased core temperature, rectal temperature, and respiration rate, and decreased vascular cross‐sectional area, blood flow, and heart rate are common clinical symptoms of increased vasoconstriction in cattle consuming ergot alkaloids from toxic endophyte‐infected tall fescue (Aiken et al., 2007, 2009; Al‐Haidary et al., 2001; Koontz et al., 2012; Rhodes et al., 1991). Prolonged display of clinical symptoms in response to fescue toxicosis could potentially manifest into impaired cardiovascular function and thermoregulation, peripheral tissue necrosis, and intrauterine growth restriction (Duckett et al., 2014; Strickland et al., 2011). Mechanisms describing how ergot alkaloid ingestion relates to the development of clinical symptoms of fescue toxicosis are not completely understood. Ergot alkaloids are structurally similar to monoamine neurotransmitters such as serotonin (5‐hydroxytryptamine; 5‐HT), dopamine, epinephrine, and norepinephrine (Berde, 1980), and thus can interact with their respective receptors to cause numerous effects on animal physiology and metabolic function (Klotz, 2015).

There are 14 distinct 5‐HT receptor subtypes (Hoyer et al., 1994) that mediate several physiological functions including appetite, behavior, energy balance, gastrointestinal secretion and motility, and vascular tone (Donovan & Tecott, 2013; El‐Merahbi et al., 2015; Yabut et al., 2019). Six of the 5‐HT receptor subtypes (5‐HT_1B_, 5‐HT_1D_, 5‐HT_2A_, 5‐HT_2B_, 5‐HT_4_, and 5‐HT_7_) have been previously reported to be vasoactive in several animal species and different blood vessel types (Snider et al., 2018). In humans, 5‐HT_1_ and 5‐HT_2_ receptor subtypes were identified as mediators of vascular contraction in isolated coronary arteries (Bax et al., 1993; Chester et al., 1990; Connor et al., 1989; Nilsson et al., 1999) and femoral veins (Glusal & Müller‐Schweinitzer, 1993). Similarly, the 5‐HT receptor subtype 5‐HT_2A_ is the predominant serotonergic receptor involved in vascular contraction of gastrointestinal, umbilical, and peripheral blood vessels in cattle and sheep (Klotz et al., 2012, 2022; Snider et al., 2018). Dietary consumption of ergot alkaloids in toxic endophyte‐infected tall fescue has been shown to decrease vascular contractile responses to individual ergopeptine alkaloids, 5‐HT, and agonists selective for the 5‐HT_2A_ receptor in isolated bovine lateral saphenous veins and mesenteric blood vessels (Egert et al., 2014; Klotz et al., 2010, 2012, 2013, 2016, 2018). Additionally, increasing exposure of the isolated bovine lateral saphenous vein to ergovaline in vitro results in increased tissue concentrations of ergovaline, suggesting bioaccumulation of ergot alkaloids occurs in vascular tissue (Klotz et al., 2009; Valente et al., 2021). Ergot alkaloids can act as both agonists and antagonists of 5‐HT_2A_‐mediated vasoconstriction (Klotz et al., 2016, 2018; Trotta et al., 2018), depending on the dose and duration of ergot alkaloid exposure (Trotta et al., 2023). Increasing 5‐HT exposure after a prior instance of ergot alkaloid exposure results in vasorelaxation of the mesenteric vein in cattle, suggesting that 5‐HT receptor subtypes other than 5‐HT_2A_ might mediate vasorelaxation in response to 5‐HT (Trotta et al., 2023).

It is well‐established that vascular contraction and relaxation responses to 5‐HT can vary depending on the animal species, type of blood vessel, and presence of 5‐HT receptor subtypes (Ni & Watts, 2006). A common finding between studies is that 5‐HT can mediate vasorelaxation in isolated veins but not in isolated arteries, unless the arteries are pre‐contracted and receptors mediating contractile responses are pharmacologically antagonized (Davis et al., 2012). Vasorelaxation of isolated veins in response to 5‐HT has been demonstrated in numerous species and across different tissues (Chand, 1981; Cocks & Arnold, 1992; Gupta, 1992; Ishine et al., 2000; Leung et al., 1996; Seitz et al., 2017; Trevethick et al., 1984; Tsuru et al., 1998; Zhang et al., 1995). Serotonin‐mediated vasorelaxation of the isolated feline saphenous vein has been previously demonstrated (Feniuk et al., 1983). However, it is currently unknown which 5‐HT receptor(s) mediate vasorelaxation in the isolated bovine lateral saphenous vein (Watts, 2016). The objectives of the current study were to determine the relaxant potential of 5‐HT and to identify potential 5‐HT receptor subtypes mediating vasorelaxation activity in bovine lateral saphenous vein.

## 2 MATERIALS AND METHODS

No live animals were involved in this study, thus approval from the University of Kentucky Animal Care and Use Committee was not required.

### 2.1 Experimental design

Two experiments were conducted to: (1) quantify 5‐HT‐mediated vasorelaxation and determine which receptor(s) were involved in 5‐HT‐mediated vasorelaxation using selective 5‐HT receptor agonists and (2) confirm the involvement of identified receptors in 5‐HT‐mediated vasorelaxation using 5‐HT receptor antagonist(s).

### 2.2 Blood vessel collection

Cranial branches of the lateral saphenous vein were collected (Klotz et al., 2006) from the left and right hind legs of six steers for Experiment 1 (body weight = 507 ± 59.5 kg) and four heifers for Experiment 2 (body weight = 650 ± 52.0 kg) immediately after slaughter at the University of Kentucky Meats Laboratory. Steers used in Experiment 1 were predominantly of Angus breeding (*n* = 5) and one steer was Holstein. The heifers used in Experiment 2 were predominantly of Angus breeding. Blood vessels were collected over a 22‐day period in Experiment 1 and a 16‐day period in Experiment 2. Collected vessel samples with surrounding adipose and connective tissues were immersed in oxygenated modified Krebs–Henseleit buffer (95% O_2_/5% CO_2_; pH = 7.4; 11.1 mM d‐glucose; 1.2 mM MgSO_4_; 1.2 mM KH_2_PO_4_; 4.7 mM KCl; 118.1 mM NaCl; 3.4 mM CaCl_2_; 24.9 mM NaHCO_3_; Sigma‐Aldrich, St. Louis, MO, USA), stored on ice, and transported to the laboratory. Blood vessels were dissected, and perivascular adipose and connective tissues were removed. Cleaned vessel segments were sliced into approximately 2‐mm cross‐sections using an adjustable acrylic tissue matrix (Braintree Scientific, 2006). Vascular dimensions (length, inner diameter, outer diameter) for vein cross‐sections were examined using a dissection microscope (Stemi 2000‐C, Carl Zeiss Inc., Oberkochen, Germany) at 12.5× magnification and recorded using Axiovision software (version 20, Carl Zeiss Inc.). In addition, vessel cross‐sections were inspected under magnification for abnormalities (structural damage incurred during dissection and cleaning and the presence of venous valves and micro‐branches) and abnormal sections were discarded and replaced with viable sections.

### 2.3 Standard preparations

For Experiment 1, stock solutions (w/v) of phenylephrine hydrochloride (P6126; Sigma‐Aldrich) was prepared in H_2_O and 5‐HT hydrochloride (H9523; Sigma‐Aldrich) and 5‐HT receptor agonists were prepared in dimethyl sulfoxide (DMSO; 472301, Sigma‐Aldrich). Serotonin receptor agonists evaluated were CP 93129 dihydrochloride (5‐HT_1B_ receptor agonist; Cat. no. 1032; 1,4‐dihydro‐3‐(1,2,3,6‐tetrahydro‐4‐pyridinyl)‐5*H*‐pyrrol[3,2‐*b*]pyridin‐5‐one dihydrochloride; Tocris Bioscience, Bristol, UK), BW 723C86 hydrochloride (5‐HT_2B_ receptor agonist; Cat. no. 1059; α‐methyl‐5‐(2‐thienylmethoxy)‐1*H*‐indole‐3‐ethanamine, monohydrochloride; Tocris Bioscience), BIMU 8 (5‐HT_4_ receptor agonist; Cat. no 4374; 2,3‐dihydro‐*N*‐[(3‐*endo*)‐8‐methyl‐8‐azabicyclo[3.2.1]oct‐3‐yl]‐3‐(1‐methylethyl)‐2‐oxo‐1*H*‐benzimidazole‐1‐carboxamide hydrochloride; Tocris Bioscience), and LP 44 (5‐HT_7_ receptor agonist; Cat. no. 2534; 4‐[2‐(methylthio)phenyl]‐*N*‐(1,2,3,4‐tetrahydro‐1‐naphthalenyl)‐1‐piperazinehexanamide, monohydrochloride; Tocris Bioscience). Initially, 5‐HT receptor agonists selective for 5‐HT_2A_ (TCB‐2; Cat. no. 2592; 4‐bromo‐3,6‐dimethoxybenzocyclobuten‐1‐yl)methylamine hydrobromide; Tocris Bioscience) and 5‐HT_1D_ (L‐694,247; Cat. no. 0781; *N*‐[4‐[[5‐[3‐(2‐aminoethyl)‐1*H*‐indol‐5‐yl]‐1,2,4‐oxadiazol‐3‐yl]methyl]phenyl]‐methanesulfonamide; Tocris Bioscience) were evaluated. However, these compounds were not further evaluated after only positive vasoactive responses (contraction) were observed (data not shown). Serial dilutions of 5‐HT and 5‐HT receptor agonist stock solutions were prepared daily to achieve desired working concentrations, ranging from 1 × 10^−9^ M to 1 × 10^−4^ M. Diluted standards were added to the incubation buffer in 25‐μL aliquots to maintain the solvent (DMSO) amount below 0.5% of the total volume and to achieve the working concentrations in myograph chambers.

For Experiment 2, stock solutions of phenylephrine hydrochloride and 5‐HT hydrochloride were prepared and diluted as described for Experiment 1. In addition, stock solutions of GR 125487 sulfamate (5‐HT_4_ receptor antagonist; Cat. no. 1658; 5‐fluoro‐2‐methoxy‐[1‐[2‐[(methylsulfonyl)amino]ethyl]‐4‐piperidinyl]‐1*H*‐indole‐3‐methylcarboxylate sulfamate; Tocris Bioscience) were prepared in DMSO. Dilution of the GR 125487 sulfamate stock solution was prepared daily to achieve desired working concentration of 1 × 10^−5^ M. Diluted standards were added to the incubation buffer in 25‐μL aliquots to achieve the working concentrations in myograph chambers.

### 2.4 Myography

Three multi‐myograph systems (DMT 610M, Danish Myo Technology, Atlanta, GA) containing four individual chambers per myograph were used for each experiment. Prior to calibration, each myograph was turned on to reach the desired temperature setting (37°C). Each chamber of the myograph was calibrated prior to the initiation of the experiment according to the manufacturer's instructions. Blood vessels were mounted onto luminal pins (Wenceslau et al., 2021) and submerged with 5 mL of Krebs–Henseleit buffer and constant gassing (95% O_2_/5% CO_2_; pH = 7.4; 37°C). The Krebs–Henseleit buffer used in myograph chambers was the same composition of the transport buffer with added 3 × 10^−5^ M desipramine (D3900; Sigma‐Aldrich) to inactivate neuronal catecholamine reuptake. An equilibration period was conducted under the conditions previously described for 90 min with buffer changes every 15 min to allow blood vessels to reach a resting tension of approximately 1 g. At completion of the 90‐min equilibration period, 25 μL of 1 × 10^−4^ M phenylephrine was added to each myograph chamber to pre‐contract each vessel segment for 15 min. Following the phenylephrine pre‐contraction, 25 μL of 5‐HT or selective 5‐HT receptor agonists were added to myograph chambers non‐cumulatively for 5 min in duplicate for each working concentration.

For Experiment 2, the myography procedure was modified as follows. At completion of the 90‐min equilibration period, 25 μL of DMSO or GR 125487 sulfamate (1 × 10^−5^ M) was added to myograph chambers for 5‐min. Then, 25 μL of 1 × 10^−4^ M phenylephrine was added to each myograph chamber to pre‐contract each vessel segment for 10 min. Following the phenylephrine pre‐contraction, 25 μL of 5‐HT was added to myograph chambers non‐cumulatively for 5 min in duplicate for each working concentration. Standard additions were added to myograph chambers non‐cumulatively to avoid potential receptor desensitization (Wenceslau et al., 2021) and to eliminate time as a factor influencing relaxation responses.

### 2.5 Vasoactive response and potency measurements

**FIGURE 1:**
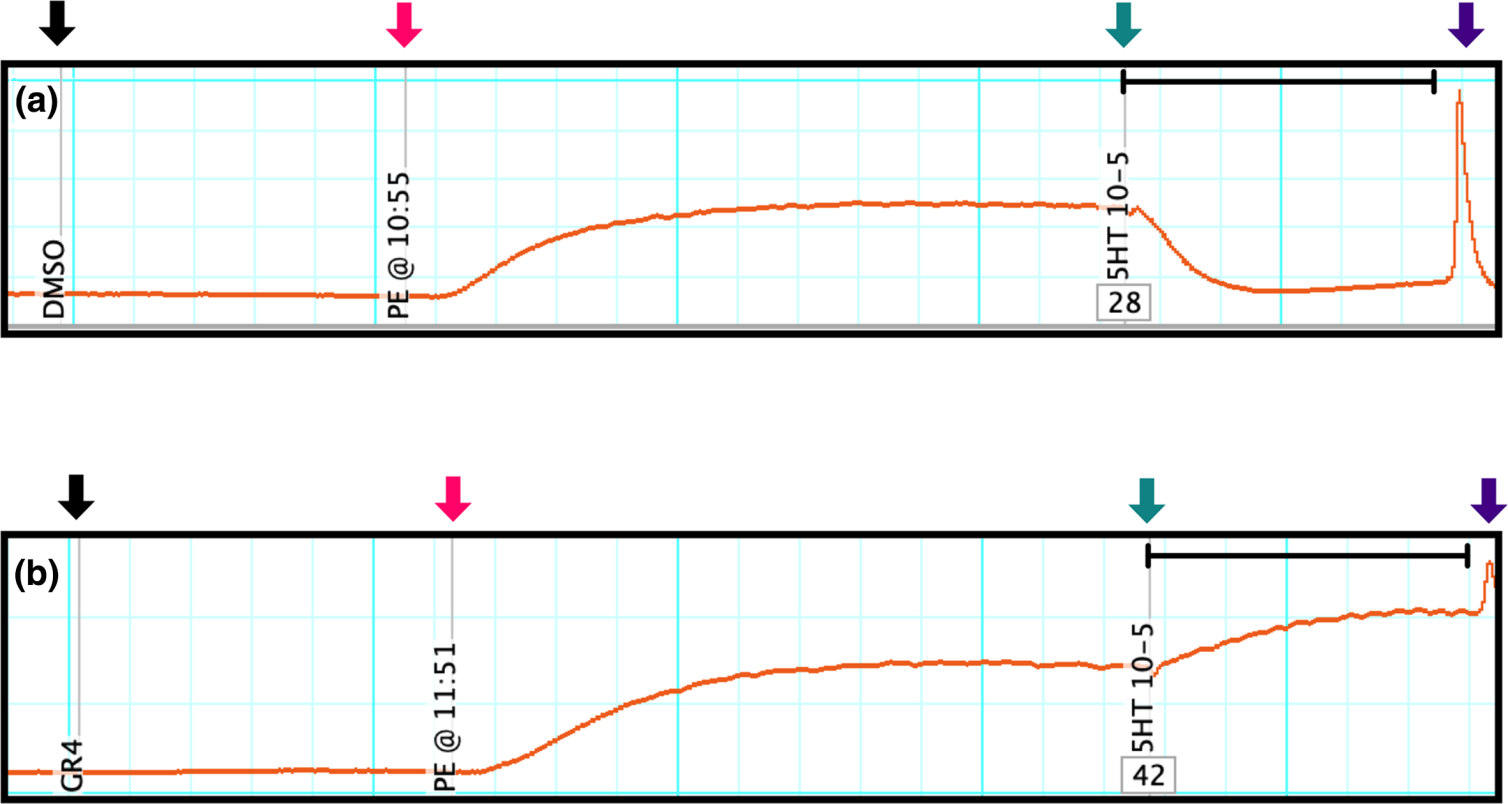
Representative trace of tension in grams from Experiment 2. Downward black arrows indicate the 25 μL addition of (a) dimethyl sulfoxide (DMSO) or (b) selective serotonin receptor 5‐HT_4_ antagonist [5‐HT_4_(‐); GR 125487 sulfamate]. Downward magenta arrows indicate when baseline tension was recorded and the 25 μL addition of 1 × 10^−4^ M phenylephrine to pre‐contract the lateral saphenous vein. Downward teal arrows indicate when the maximum grams of tension in response to 1 × 10^−4^ M phenylephrine pre‐contraction was measured and when the 25 μL addition of 5‐HT occurred. Downward purple arrows indicate the end of the 5‐min agonist exposure period and when the buffer was evacuated from myograph chambers. The area in between the downward teal and purple arrows is indicated by a black capped line and is where the vasoactive response was measured. When the vasoactive response was lesser than the phenylephrine pre‐contraction maximum tension (a), the minimum grams of tension was measured. When the vasoactive response was greater than the phenylephrine pre‐contraction maximum tension (b), the maximum grams of tension was measured.

Vasoactive responses to treatments were measured for each animal in duplicate and averaged. Isometric changes in tension (measured in millinewtons) of lateral saphenous vein segments in response to phenylephrine, 5‐HT, or 5‐HT receptor agonists were digitized and converted to mass units (in grams) using PowerLab16/35 and Chart software (version 8.1, ADInstruments, Colorado Springs, CO). A representative trace from Experiment 2 is presented (Figure 1). Baseline tension was determined just prior to the addition of 1 × 10^−4^ M phenylephrine. The tension (measured in grams) during the phenylephrine pre‐contraction period was determined after plateau (15 min for Experiment 1 and 10 min for Experiment 2) and corrected for baseline tension (Wenceslau et al., 2021). If the greatest change in tension during the 5‐min treatment incubation period was increased compared to the pre‐contraction maximum, the maximum grams of tension was measured. If the greatest change in tension during the 5‐min treatment incubation period was decreased compared to the pre‐contraction maximum, the minimum grams of tension was measured. Recorded grams of tension during the 5‐min treatment incubation period was normalized as a percentage of the maximum grams of tension induced by the phenylephrine pre‐contraction at plateau to compensate for differences in vessel responsiveness. Vessel vasoactive response data were reported as the percent mean vasoactive response (±standard error of the mean) of the maximum contractile response produced by the phenylephrine pre‐contraction. A vasoactive response greater than 100% was considered a contraction and a vasoactive response less than 100% was considered a relaxation. Vasoactive response data were plotted using nonlinear regression with fixed slope (GraphPad Prism 9.5; Dotmatics, Boston, MA) and calculated using a log(agonist) vs. response three parameter equation (Wenceslau et al., 2021):

$$y=\text{bottom}+\frac{\left(\mathrm{top}-\text{bottom}\right)}{\left[1+10^{\left(x-\text{logEC}50\right)}\right]}$$

where *y* represents vasoactive response, *x* represents 5‐HT or 5‐HT receptor agonist concentration, top and bottom are plateaus in units of the *y*‐axis (percentage of phenylephrine pre‐contraction), and 50% effective concentration (EC_50_) is the molar concentration of 5‐HT or 5‐HT receptor agonist producing 50% of the response between plateaus.

### 2.6 Statistical analysis

For Experiment 1, all vasoactive response data were analyzed separately for each compound evaluated using SAS (version 9.4; SAS Institute Inc., Cary, NC). Vasoactive response data were analyzed as a completely randomized design using the GLM procedure for the fixed effect of agonist concentration. For Experiment 2, vasoactive response data were analyzed as a completely randomized design using the GLM procedure for fixed effects of 5‐HT_4_ receptor antagonist concentration, 5‐HT concentration, and the 5‐HT_4_ receptor antagonist × 5‐HT concentration interaction. Least squares means and their standard errors were computed for each fixed effect included in the models. Because of the variable sample size, standard error of the mean (SEM) was used to illustrate the uncertainty surrounding the means. Pairwise comparisons of least squares means were separated using the Tukey–Kramer adjustment, protected by a significant *F*‐test. Results were considered significant if *p* ≤ 0.05.

## 3 RESULTS

### 3.1 Experiment 1: Characterization of 5‐HT‐mediated vasoactivity and 5‐HT receptor‐mediated vasorelaxation in the isolated saphenous vein

**FIGURE 2:**
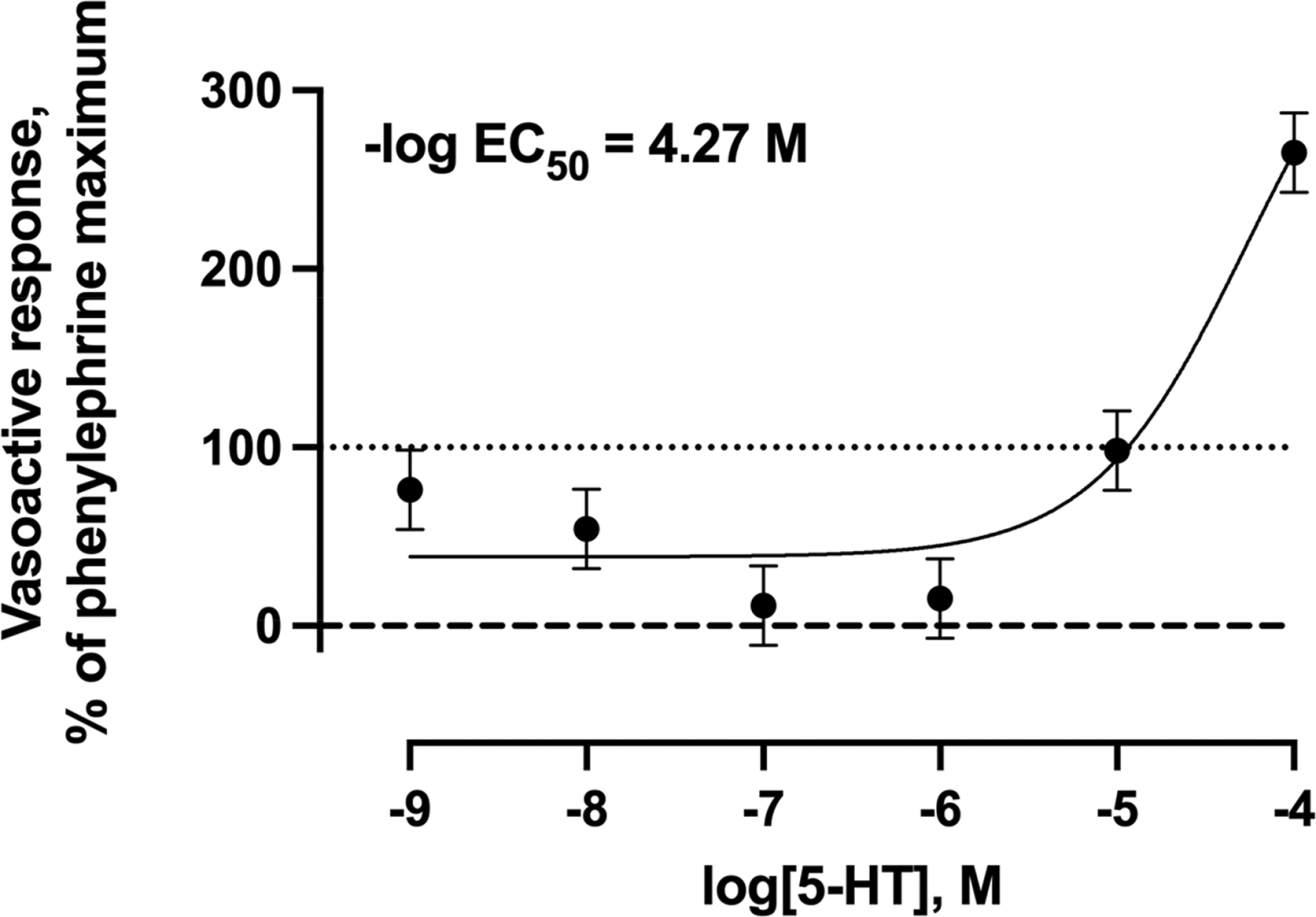
Least squares mean vasoactive response ± SEM (*n* = 6) of bovine lateral saphenous vein to non‐cumulative increasing concentrations of serotonin (5‐HT) after pre‐contraction with 1 × 10^−4^ M phenylephrine. The dotted line at *y* = 100 represents the normalized vasoactive response to the phenylephrine pre‐contraction. The dashed line at *y* = 0 represents the normalized vasoactive response at baseline tension. Probability value for 5‐HT concentration: *p* < 0.001.

The length, inner diameter, and outer diameter of the lateral saphenous vein cross‐sections were 2.59 ± 0.31 mm, 0.829 ± 0.180 mm, and 3.12 ± 0.29 mm, respectively. Increasing concentrations of 5‐HT resulted in divergent vasoconstriction and vasorelaxation responses that were dose‐dependent (*p* < 0.001; Figure 2). As 5‐HT concentrations increased from 1 × 10^−7^ M to 1 × 10^−6^ M, the vasoactive response decreased by 88.7% and 84.7% below the phenylephrine pre‐contraction maximum (relaxation). As 5‐HT concentrations increased from 1 × 10^−6^ M 5‐HT to 1 × 10^−5^ M 5‐HT, the vasoactive response increased, returning the vasoactive response to the phenylephrine pre‐contraction maximum. At 1 × 10^−4^ M 5‐HT, the vasoactive response increased by 165% above the phenylephrine pre‐contraction maximum (contraction).

**FIGURE 3:**
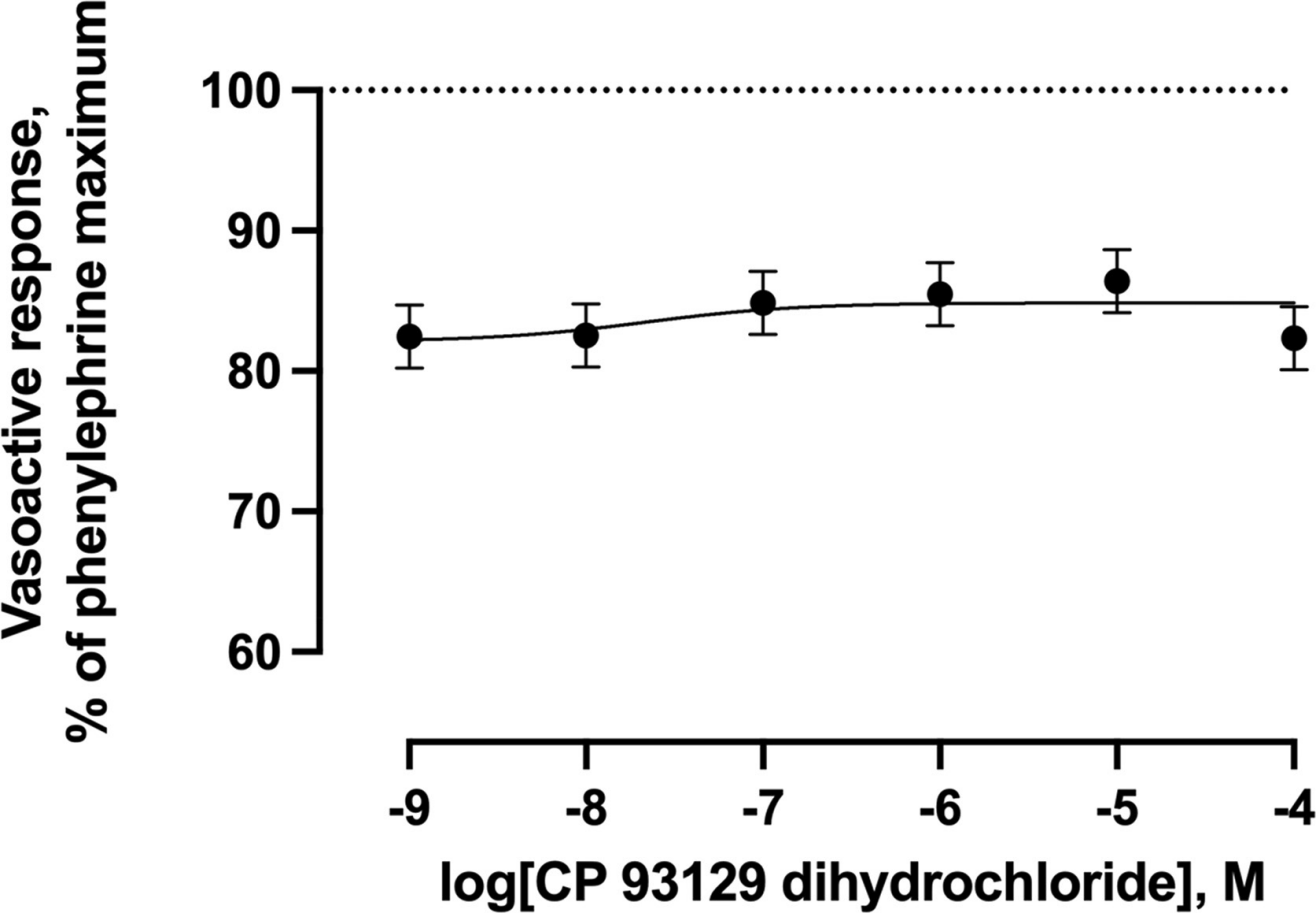
Least squares mean vasoactive response ± SEM (*n* = 6) of bovine lateral saphenous vein to non‐cumulative increasing concentrations of selective 5‐HT_1B_ receptor agonist (CP 93129 dihydrochloride) after pre‐contraction with 1 × 10^−4^ M phenylephrine. The −log EC_50_ was not reported due to the lack of effect of CP 932129 concentration on vasoactive response (*p* > 0.05). The dotted line at *y* = 100 represents the normalized vasoactive response to the phenylephrine pre‐contraction. *Y*‐axis units do not begin at 0. Probability value for agonist concentration: *p* = 0.69.

**FIGURE 4:**
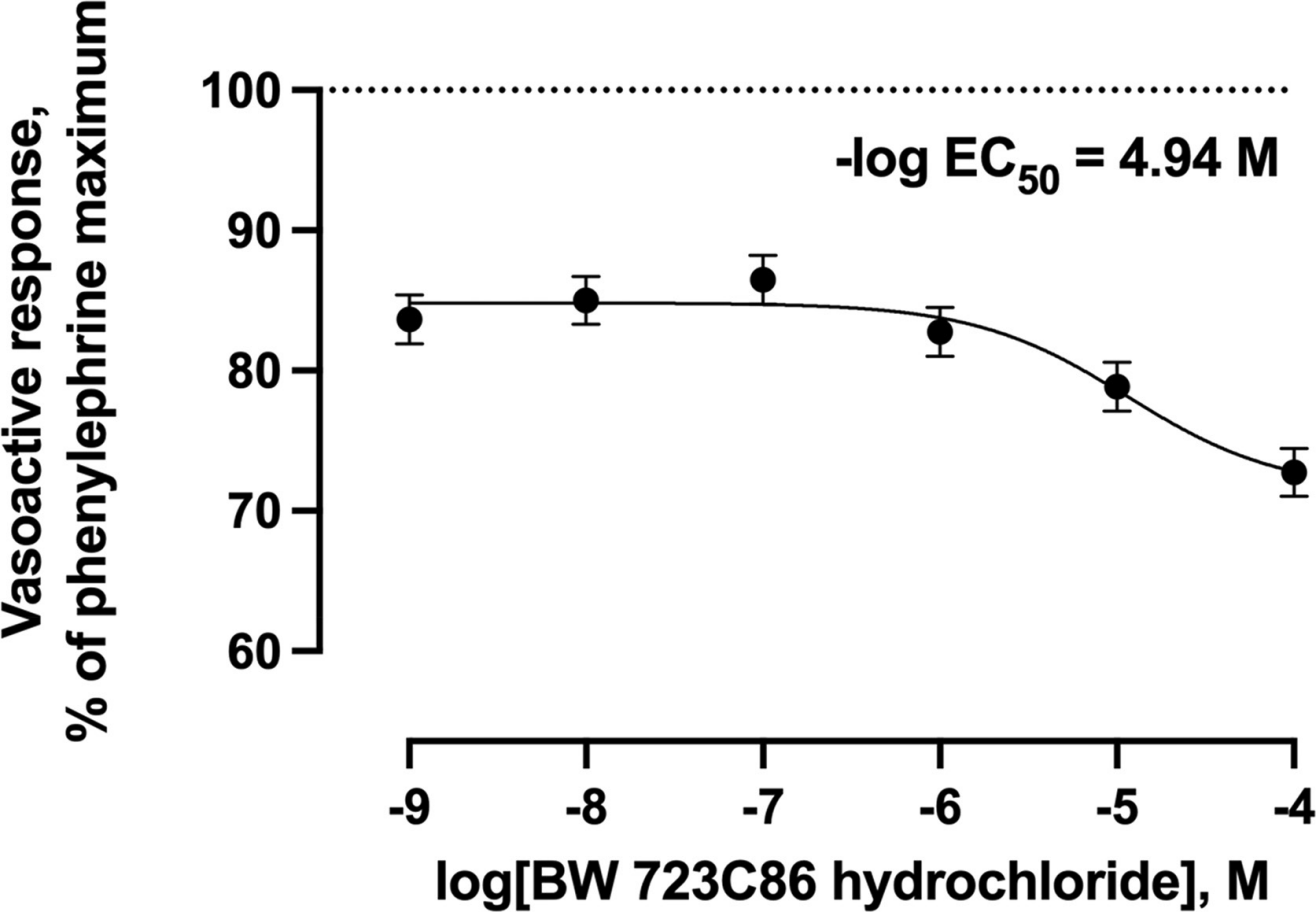
Least squares mean vasoactive response ± SEM (*n* = 6) of bovine lateral saphenous vein to non‐cumulative increasing concentrations of selective 5‐HT_2B_ receptor agonist (BW 723C86 hydrochloride) after pre‐contraction with 1 × 10^−4^ M phenylephrine. The dotted line at *y* = 100 represents the normalized vasoactive response to the phenylephrine pre‐contraction. *Y*‐axis units do not begin at 0. Probability value for agonist concentration: *p* < 0.001.

**FIGURE 5:**
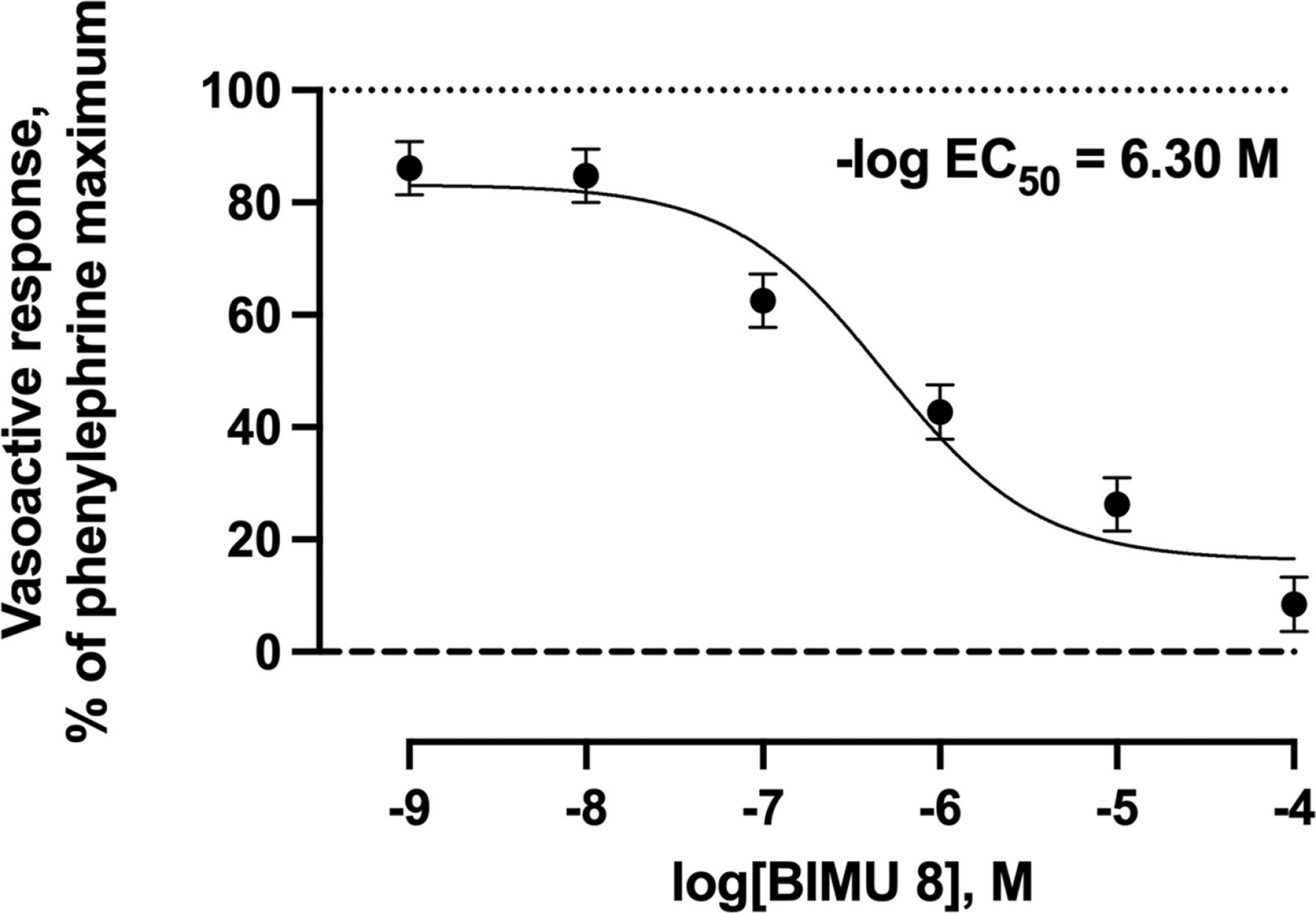
Least squares mean vasoactive response ± SEM (*n* = 5) of bovine lateral saphenous vein to non‐cumulative increasing concentrations of selective 5‐HT_4_ receptor agonist (BIMU 8) after pre‐contraction with 1 × 10^−4^ M phenylephrine. The dotted line at *y* = 100 represents the normalized vasoactive response to the phenylephrine pre‐contraction. The dashed line at *y* = 0 represents the normalized vasoactive response at baseline tension. Probability value for agonist concentration: *p* < 0.001.

**FIGURE 6:**
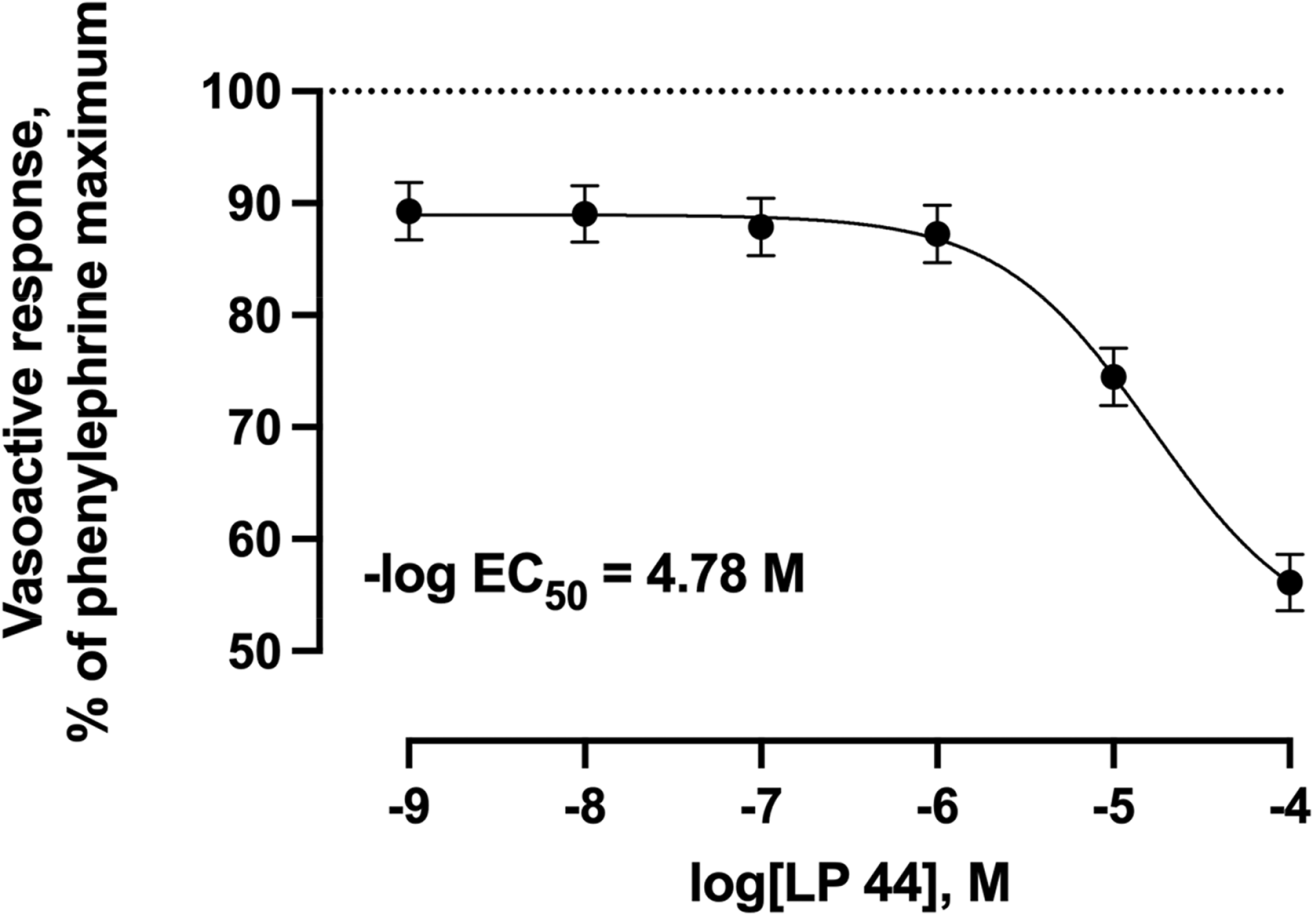
Least squares mean vasoactive response ± SEM (*n* = 5) of bovine lateral saphenous vein to non‐cumulative increasing concentrations of selective 5‐HT_7_ receptor agonist (LP 44) after pre‐contraction with 1 × 10^−4^ M phenylephrine. The dotted line at *y* = 100 represents the normalized vasoactive response to the phenylephrine pre‐contraction. *Y*‐axis units do not begin at 0. Probability value for agonist concentration: *p* < 0.001.

Increasing concentrations of the selective 5‐HT_1B_ agonist did not result (*p* = 0.69) in any vasoactive response (Figure 3). Increasing concentrations of agonists selective for 5‐HT_2B_, 5‐HT_4_, or 5‐HT_7_ receptors resulted in 27.3%, 91.6%, and 43.9% (*p* < 0.001) decreases in the vasoactive responses below the phenylephrine pre‐contraction maximums, respectively (Figures 4, 5, 6). Of these 5‐HT receptor agonists, the selective 5‐HT_4_ receptor agonist resulted in the lowest −log EC_50_ value (6.30) compared with 5‐HT_2B_ and 5‐HT_7_ receptor agonists (4.21 and 4.66, respectively) demonstrating a greater potency of inducing vasorelaxation.

### 3.2 Experiment 2: 5‐HT‐mediated vasoactivity in the absence and presence of the 5‐HT_4_ receptor antagonist

**FIGURE 7:**
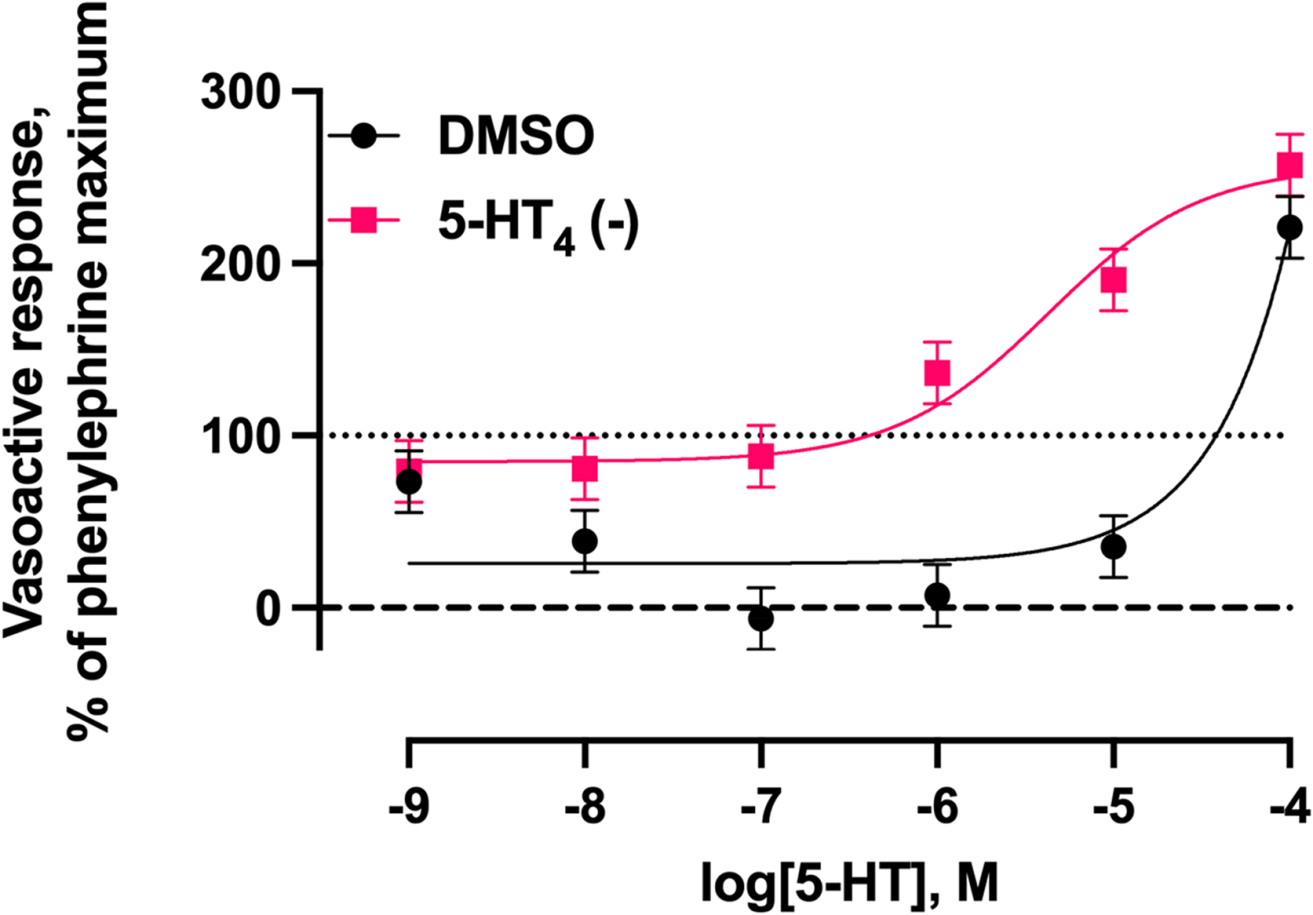
Least squares mean vasoactive response ± SEM (*n* = 4) of bovine lateral saphenous vein to non‐cumulative increasing concentrations of 5‐HT in the presence of dimethyl sulfoxide (DMSO) or a selective serotonin receptor 5‐HT_4_ antagonist [5‐HT_4_(‐); GR 125487 sulfamate] after pre‐contraction with 1 × 10^−4^ M phenylephrine. The dotted line at *y* = 100 represents the normalized vasoactive response to the phenylephrine pre‐contraction. The dashed line at *y* = 0 represents the normalized vasoactive response at baseline tension. Probability values for 5‐HT_4_ antagonist, 5‐HT concentration, and the 5‐HT_4_ antagonist × 5‐HT concentration interaction: *p* < 0.001.

The length, inner diameter, and outer diameter of the lateral saphenous vein cross‐sections were 2.61 ± 0.154 mm, 0.733 ± 0.140 mm, and 3.09 ± 0.206 mm, respectively. There was a 5‐HT_4_ receptor antagonist × 5‐HT concentration interaction (*p* < 0.001) for the vasoactive response of the lateral saphenous vein (Figure 7). At 1 × 10^−9^ M 5‐HT, the vasoactive response did not differ among treatments (with or without the antagonist). As 5‐HT concentration increased from 1 × 10^−8^ M to 1 × 10^−5^ M, there was a divergent vasoactive response. Increasing 5‐HT concentrations decreased the vasoactive response below the phenylephrine pre‐contraction maximum in the absence of the 5‐HT_4_ antagonist. In the presence of the 5‐HT_4_ antagonist, the decrease in the vasoactive response below the phenylephrine pre‐contraction maximum was attenuated and increases in the vasoactive response above the phenylephrine pre‐contraction maximum were observed at 1 × 10^−6^ M and 1 × 10^−5^ M 5‐HT. At 1 × 10^−4^ M 5‐HT, an increase in the vasoactive response above the phenylephrine pre‐contraction maximum was observed in the absence and presence of the 5‐HT_4_ antagonist and the magnitude of the response did not differ between treatments at this concentration of 5‐HT.

## 4 DISCUSSION

### 4.1 5‐HT stimulates concentration‐dependent vasoconstriction and vasorelaxation in the isolated saphenous vein

Early studies identified that 5‐HT isolated from porcine and bovine serum functioned as a hypertensive agent and could change vascular tone (Rapport et al., 1948; Watts, 2016; Whitaker‐Azmitia, 1999). Several studies have demonstrated that 5‐HT causes a concentration‐dependent contraction in isolated arteries and veins in multiple species (Page & McCubbin, 1953; Watts et al., 2012). Under most circumstances, 5‐HT‐induced vasoconstriction is mediated primarily through 5‐HT_2A_ but mediation could also occur through 5‐HT_1B_, 5‐HT_1D_, or 5‐HT_2B_ during hypertension (Banes & Watts, 2002; Klotz et al., 2018; Russell et al., 2002). In the current study, increased concentrations of 5‐HT induced a contractile response greater than the pre‐contracted saphenous vein. This is similar to prior studies in cattle saphenous vein that evaluated 5‐HT without a pre‐contraction period (Klotz et al., 2012, 2013; Valente et al., 2021). Notably, the 5‐HT concentration curve of the bovine lateral saphenous vein of the current study appears to be similar to the response observed by Watts (Watts, 2016) in the pre‐contracted rat tail artery in the presence of 5‐HT_2A_ and 5‐HT_1B_ antagonists. In both studies, 5‐HT causes a concentration‐dependent relaxation as 5‐HT concentrations increased to 1 × 10^−6^ M. At concentrations greater than 1 × 10^−6^ M, 5‐HT caused a vascular contraction. These studies provide greater insight into the dual nature of 5‐HT as a vasoconstrictor and vasodilator in isolated blood vessels.

Numerous previous studies have demonstrated that 5‐HT stimulated vasorelaxation of isolated veins in different species including cat saphenous vein (Feniuk et al., 1983), sheep pulmonary vein (Cocks & Arnold, 1992; Zhang et al., 1995), goat pulmonary vein (Chand, 1981), guinea pig jugular vein (Gupta, 1992), monkey jugular vein (Leung et al., 1996), pig pial vein (Ishine et al., 2000), pig vena cava (Trevethick et al., 1984), rabbit facial vein (Tsuru et al., 1998), rat mesenteric vein (Watts et al., 2015), rat hepatic portal vein (Seitz et al., 2017), and bovine mesenteric vein (Trotta et al., 2023). To our knowledge, this is the first study which has demonstrated that 5‐HT causes vasorelaxation of the isolated bovine saphenous vein. Interestingly, maximal vasorelaxation activity in response to 5‐HT was observed in the range of 1 × 10^−7^ M to 1 × 10^−6^ M. Circulating 5‐HT concentrations are typically within this same range in cattle (Moore et al., 2015; Valente et al., 2021), suggesting that 5‐HT doses which resulted in vasorelaxation were physiologically relevant to the bovine model. However, it remains unknown how increasing circulating 5‐HT concentration in cattle influences vasodilation in vivo.

### 4.2 5‐HT‐mediated vasorelaxation in the isolated saphenous vein occurs through 5‐HT_4_ activation

In Experiment 1, 5‐HT receptor agonists that were highly selective for 5‐HT_1B_ [CP 93129 dihydrochloride (Hamid et al., 2014; Koe et al., 1992; Lee et al., 1998)], 5‐HT_2B_ [BW 723C86 hydrochloride (Duxon et al., 1997; Kennett et al., 1997; Niebert et al., 2011)], 5‐HT_4_ [BIMU 8 (Pellissier et al., 2009; Schaus et al., 1998)], and 5‐HT_7_ [LP 44 (Leopoldo et al., 2004)] were evaluated for their effects on vasoactivity in the isolated bovine lateral saphenous vein. Of the 5‐HT receptor agonists evaluated, 5‐HT_2B_, 5‐HT_4_, and 5‐HT_7_ receptor activation resulted in vasorelaxation of the bovine saphenous vein. Under the conditions of the current study, the 5‐HT_4_ receptor agonist displayed the lowest −log EC_50_ value (6.30) compared with 5‐HT_2B_ and 5‐HT_7_ receptor agonists (4.21 and 4.66, respectively) in the isolated bovine lateral saphenous vein. These results are interpreted to suggest that vasorelaxation via 5‐HT_4_ activation occurs at a lesser concentration compared with 5‐HT_2B_ or 5‐HT_7_ activation. Maximal vasorelaxation activity was observed at a concentration of 1 × 10^−4^ M for 5‐HT_2B_, 5‐HT_4_, and 5‐HT_7_ receptor agonists. However, the magnitude of the decrease in the vasoactive response from the phenylephrine maximum occurring at 1 × 10^−4^ M was much greater for the 5‐HT_4_ receptor agonist (92% decrease below the phenylephrine pre‐contraction maximum) compared with 5‐HT_2B_ (27% decrease below the phenylephrine pre‐contraction maximum) or 5‐HT_7_ receptor agonists (44% decrease below the phenylephrine pre‐contraction maximum). Therefore, this experiment provided strong evidence that 5‐HT_4_ is likely the primary receptor mediating vasorelaxation in response to 5‐HT observed in the bovine lateral saphenous vein.

In Experiment 2, increasing 5‐HT caused a dose‐dependent relaxation of the isolated saphenous vein in the absence of the 5‐HT_4_ receptor antagonist. In the presence of the 5‐HT_4_ receptor antagonist, nearly all 5‐HT‐mediated vasorelaxation activity was attenuated. Maximal vasorelaxation was observed at 1 × 10^−7^ M 5‐HT and 94% of 5‐HT‐mediated vasorelaxation could be accounted for through 5‐HT_4_. Therefore, this experiment confirmed that 5‐HT‐mediated vasorelaxation occurs primarily through 5‐HT_4_ activation in the isolated bovine saphenous vein.

Compared with other 5‐HT receptor subtypes, there is less information on the role of 5‐HT_4_ in cardiovascular function. The 5‐HT_4_ receptor is a seven transmembrane spanning G protein‐coupled receptor that exists in two complexes, one coupling G_s_ and one coupling G_i_ proteins (Huang et al., 2022). Several prior investigations into the functional roles of 5‐HT_4_ described its function in tissues of the central nervous system, gastrointestinal tract, bladder, heart, and adrenal gland (Hegde & Eglen, 1996). Enterokinetic drugs such as prucalopride (Shin et al., 2014; Shokrollahi et al., 2019; Wong et al., 2010), metoclopramide (Bockaert et al., 1990; Elswood et al., 1991; Elz & Keller, 1995), and zacopride (Bhandari & Andrews, 1991; Gullikson et al., 1992) can function as full or partial 5‐HT_4_ receptor agonists, altering gastrointestinal motility to alleviate chronic constipation and prevent nausea. Notably, Cocks and Arnold (Cocks & Arnold, 1992) were the first to demonstrate vasorelaxation via 5‐HT_4_ receptor activation in the isolated ovine pulmonary vein. In their study, 5‐HT_4_‐mediated vasorelaxation appeared to be specific to ruminants because there was no evidence for 5‐HT_4_‐mediated vasorelaxations of the dog, pig, or human pulmonary vein (Cocks & Arnold, 1992). Other studies with isolated ovine or caprine pulmonary vein have demonstrated 5‐HT‐induced vasorelaxation but, the involvement of 5‐HT_4_ was not evaluated (Chand, 1981; Eyre, 1975; Zhang et al., 1995). The results of the current study support the concept that 5‐HT_4_‐mediated vasorelaxation may be ruminant‐specific; however, it remains unknown if the isolated saphenous vein from nonruminant species would relax to 5‐HT via 5‐HT_4_ receptor activation.

There is a lack of clear evidence describing the mechanism by which 5‐HT_4_ receptor activation leads to relaxation of vascular tissue. Cocks and Arnold (1992) demonstrated that 5‐HT_4_‐mediated vasorelaxation was endothelium‐independent in the isolated ovine pulmonary vein. A later study demonstrated that 5‐HT‐induced vasorelaxation of the isolated ovine pulmonary vein increased tissue cyclic adenosine monophosphate (cAMP) concentrations and suggested that cAMP was involved in secondary messenger signaling (Zhang et al., 1995). This finding is supported by positive coupling of the 5‐HT_4_ receptor to adenylate cyclase in other tissues (Bockaert et al., 1990; Dumuis et al., 1988). Further research is necessary to describe the signaling mechanism associated with 5‐HT_4_‐mediated vasorelaxation of vascular tissue in ruminants.

In conclusion, 5‐HT causes both concentration‐dependent vasoconstriction and vasorelaxation in the isolated bovine lateral saphenous vein. Vasorelaxation of the bovine saphenous vein can be partially mediated through 5‐HT_2B_, 5‐HT_4_, and 5‐HT_7_ receptors. Approximately 94% of the maximal vasorelaxation activity occurring in response to 5‐HT could be accounted for through 5‐HT_4_, providing strong evidence that 5‐HT‐mediated vasorelaxation occurs primarily through 5‐HT_4_ receptor activation in bovine lateral saphenous vein. The isolated bovine lateral saphenous vein is an excellent model to further study the functional role of the 5‐HT_4_ receptor in vascular tissue and signaling mechanisms associated with 5‐HT_4_ receptor activation. A greater understanding of receptor‐mediated vasorelaxation mechanisms could potentially lead to the development of strategies to mitigate sustained ergot alkaloid induced vasoconstriction that occurs during fescue toxicosis in cattle.

## AUTHOR CONTRIBUTIONS

R.J.T. and J.L.K. conceived and designed the research; R.J.T. and J.L.K. performed the experiments; R.J.T. analyzed the data; R.J.T., D.L.H., and J.L.K. interpreted results of the experiments; R.J.T. prepared the figures; R.J.T. drafted the manuscript; R.J.T., D.L.H., and J.L.K. edited and revised the manuscript; R.J.T., D.L.H., and J.L.K. approved final version of the manuscript.

## FUNDING INFORMATION

Non‐Assistance Cooperative Agreement, USDA‐ARS Forage‐Animal Production Research Unit and University of Kentucky Agricultural Experiment Station, Grant/Award Number: 5042‐32630‐004‐001‐S (to DLH); USDA‐ARS National Program 101—Food Animal Production, Grant/Award Number: 5042‐32630‐004‐000‐D (to JLK).

## CONFLICT OF INTEREST STATEMENT

The authors declare that there are no conflicts of interest.

## ETHICS STATEMENT

No live animals were involved in this study, thus approval from the University of Kentucky Animal Care and Use Committee was not required.

## DISCLAIMERS

Mention of trade name, proprietary product of specified equipment does not constitute a guarantee or warranty by the USDA and does not imply approval to the exclusion of other products that may be available.

## Data Availability

Data are available upon reasonable request to the corresponding author.
